# Commonalities and Disparities between Endometriosis and Chronic Endometritis: Therapeutic Potential of Novel Antibiotic Treatment Strategy against Ectopic Endometrium

**DOI:** 10.3390/ijms24032059

**Published:** 2023-01-20

**Authors:** Kotaro Kitaya, Tadahiro Yasuo

**Affiliations:** 1 Infertility Center, Kouseikai Mihara Hospital/Katsura Mihara Clinic, 6-8, Kamikatsura Miyanogo-cho, Nishikyo-ku, Kyoto 615-8227, Japan; 2 Department of Obstetrics and Gynecology, Otsu City Hospital, Otsu 520-0804, Japan

**Keywords:** chronic endometriosis, endometriosis, in vitro fertilization, metronidazole, repeated implantation failure

## Abstract

Chronic endometritis (CE) is a local mucosal inflammatory disorder of the uterine lining, which is histopathologically recognized as the unusual infiltration of CD138(+) plasmacytes into the endometrial stromal compartment. Accumulating body of research documented that CE is associated with female infertility and several obstetric/neonatal complications. The major cause of CE is thought to be intrauterine infection represented by common bacteria (*Escherichia coli*, *Enterococcus faecalis*, *Streptococcus*, and *Staphylococcus*), *Mycoplasma/Ureaplasma*, and *Mycobacterium*. Additionally, local dysbiosis in the female reproductive tract may be involved in the onset and development of CE. Antibiotic treatments against these microorganisms are effective in the elimination of endometrial stromal plasmacytes in the affected patients. Meanwhile, endometriosis is a common female reproductive tract disease characterized by endometriotic tissues (ectopic endometrium) growing outside the uterus and potentially causes chronic pelvic symptoms (dysmenorrhea, dyspareunia, dyschezia, and dysuria), infertility, and ovarian cancers. Endometriosis involves endocrinological, genetic, and epigenetic factors in its etiology and pathogenesis. Recent studies focus on immunological, inflammatory, and infectious aspects of endometriosis and demonstrate several common characteristics between endometriosis and CE. This review aimed to better understand the immunological and microbial backgrounds underlying endometriosis and CE and look into the therapeutic potential of the novel antibiotic treatment strategy against endometriosis in light of endometrial infectious disease.

## 1. Introduction

Chronic endometritis (CE) is a local mucosal inflammatory disorder of the uterine lining [1,2,3]. Contrary to acute endometritis, which presents with intense clinical manifestations, including systemic fever, pelvic pain, and increased vaginal flow, CE is so asymptomatic/oligosymptomatic that it is often overlooked by both affected patients and experienced gynecologists [4,5]. Such nondescript clinical nature of CE has hindered research on this disease. Given that the occurrence, progress, and remission of CE remain fully undetermined, it is controversial if the term “chronic” is appropriate to describe the entity of this pathologic condition.

Accumulating body of research, however, documented that CE is frequently identified in infertile women with a history of unknown etiology (28%), repeated implantation failure following in vitro fertilization-embryo transfer (IVF-ET) cycles (14–31%), and recurrent pregnancy loss (9–13%) [4,5,6,7,8,9]. Moreover, chronic deciduitis, a potentially persistent form of CE during the gestational period, is associated with several obstetric/neonatal complications (preterm labor, pre-eclampsia, periventricular leukomalacia, and cerebral palsy in premature infants) [10,11]. The landmark of the histopathologic feature in CE is the unusual infiltration of endometrial stromal plasmacytes (ESPCs), thereby recent studies adopt immunohistochemistry for CD138, a marker of plasmacytes (IHC-CD138) [1]. The major cause of CE is thought to be intrauterine infection represented by common bacteria (*Escherichia coli*, *Enterococcus faecalis, Streptococcus, Staphylococcus, Mycoplasma/Ureaplasma,* and *Mycobacterium*) [2,3]. A few reports implicate the association of human immunodeficiency virus and cytomegalovirus in CE, although the causality between these viral infections and CE remains uncertain [4,5]. Additionally, local dysbiosis in the female reproductive tract may be involved in the onset and development of CE [6]. Antibiotic treatments against these microorganisms have been shown to effectively eradicate CD138(+) ESPCs in the affected women [4,5].

Meanwhile, endometriosis is a common female reproductive tract disease characterized by endometriotic tissues (ectopic endometrium) growing outside the uterus. The common sites of endometriosis are ovaries, fallopian tubes, and peritoneum, and ectopic endometrial tissues occasionally expand further into extrapelvic organs (rectum, umbilicus, abdominal wall, thorax, vulva, and central nervous system). Endometriosis potentially causes chronic pelvic symptoms (dysmenorrhea, dyspareunia, dyschezia, and dysuria), infertility, and ovarian cancers in the affected women. Endometriosis involves endocrinological, genetic, and epigenetic factors in its etiology and pathogenesis [12]. Recent studies focus on immunological, infectious, and inflammatory aspects of endometriosis and demonstrate several common characteristics between endometriosis and CE [13]. This review aimed to better understand the immunological and microbial backgrounds underlying endometriosis and CE and look into the therapeutic potential of the novel antibiotic treatment strategy against endometriosis in light of endometrial infectious disease.

## 2. Prevalence of CE in Women with and without Endometriosis

CE has been reported to be identified in 3–53% of patients with endometriosis (Table 1). These inter-study variances in the prevalence are attributed mainly to the differences in the diagnostic criteria that the researches adopted [the density of CD138(+) ESPCs in the unit areas in the endometrial stromal compartment and the light microscopic higher power fields observed] and the laboratory procedures for detection of CD138(+) ESPCs (the clones, dilutions, and temperatures and time for incubation of the primary antibody as well as the preparations and conditions of the endometrial specimens).

Using the archival full-thickness eutopic endometrial tissues obtained from premenopausal women undergoing hysterectomy due to benign uterine corpus disorders (fibroids, adenomyosis, endometriosis) and benign ovarian tumors, we first investigated the prevalence of CE in the histopathologic CE (defined as five or more CD138(+) ESPCs in 10 microscopic high power fields (HPFs), 400-fold magnification) in 2011. CE was identified in 11.7% of the non-endometriosis group and 5.0% of the endometriosis group [14]. However, the results of this study were inconclusive due to its retrospective nature and small sample size. In 2014, Takebayashi et al. [15] also investigated the prevalence of CE in a retrospective analysis of a larger number of archival full-thickness eutopic endometrial tissues obtained from premenopausal women undergoing hysterectomies due to benign uterine pathology. In contrast to 27.0% of women without endometriosis, CE was found in 52.9% of women with endometriosis (*p* = 0.031). This is the highest number on the prevalence of CE published to date. The high prevalence of CE in this study is likely due to the diagnostic criteria they employed (defined as one or more CD138(+) ESPCs in 10 HPFs, 400-fold magnification). However, the overall prevalence of CE was yet higher in the endometriosis group than in the non-endometriosis group (29.41% vs. 5.4%, *p* = 0.0101) even when a more stringent threshold (six or more ESPCs in one HPF) was adopted. Additionally, they discovered that all women in the endometriosis group had more than 11 ESPCs in one HPF. There were no relationships between CE and the patient demographics, such as age, body mass index (BMI), gravidity, parity, and the stage of endometriosis (according to the revised American Society for Reproductive Medicine classification) [20]. Endometriosis is often concomitant with uterine fibroids and adenomyosis. According to stepwise logistic regression analysis, there were no significant associations between CE and these two frequent uterine benign diseases, along with carcinoma in situ of the uterine cervix.

With the eutopic endometrial samples in the hysterectomized specimens, Cicinelli et al. [17] compared the prevalence of CE (one or more CD138(+) ESPCs in 10 HPFs, 100-fold magnification) in women with advanced endometriosis (stage IV according to the revised American Society for Reproductive Medicine classification) and those without endometriosis. The prevalence was significantly higher in the endometriosis group than in the non-endometriosis group (42.3% versus 15.4% according to hysteroscopy; and 38.5% versus 14.1% according to histopathology, *p* < 0.001). Again, there were no significant associations between CE and age, BMI, and the presence/absence of uterine fibroids and/or adenomyosis. Multiparity, however, was found as a factor that lowered the prevalence of CE in women with endometriosis both in univariate and multivariate analyses.

Khan et al. [16] enrolled more women undergoing diagnostic or surgical laparoscopy with endometriosis. When CE was defined as the presence of one or more CD138(+) ESPCs in 15 HPFs (100 × magnification) in three or more sections of eutopic endometrial curettage specimens obtained during the operation, its prevalence was 3.1% in the endometriosis group and 0% in the non-endometriosis group (no statistical difference). These numbers are much different from another prospective non-randomized study published later in 2021 by the same research group (endometriosis group 22.6%~ and non-endometriosis group 23.4%~) [19], despite that they employed the same sample preparation and examination models. Although the discrepancies between the two studies remain fully unexplained, they may arise from (i) the difference in the diagnostic criteria employed (one or more ESPCs in five HPFs, 200-fold magnification, in the latter study), (ii) the absence (the former study) or presence (the latter study) of histopathologic examinations for CE prior to laparoscopy, (iii) preoperative administration of the oral antibiotic agents (levofloxacin, 500 mg, once), and/or intramuscular gonadotropin-releasing hormone agonist (1.88 mg per month, three times) in the latter study, and (iv) the difference in age of the women enrolled. In this prospective study, there were no statistical differences in the prevalence of CE between the endometriosis group and the non-endometriosis group, as well as among the stages of endometriosis.

Meanwhile, Freitag et al. [18] reported the prevalence of CE in infertile women with or without endometriosis undergoing endometrial scratching in the secretory phase of the menstrual cycle before proceeding to assisted reproductive treatment cycles and those with a history of repeated implantation failure and/or recurrent pregnancy loss. Using the diagnostic criteria as five or more CD138(+) ESPCs per mm^2^ section, the prevalence of CE was similar (*p* = 0.634) between the endometriosis group and the non-endometriosis group (12.9% versus 10.0%). The bias of this study, however, was that some women (the number not detailed in the article) did not take laparoscopy or laparotomy and were regarded as having endometriosis only by symptomatology. Holzer et al. [21] included infertile women with endometriosis in the follicular phase (87%) and other phases in their prospective analysis. The prevalence of CE (defined as one or more CD138(+) ESPCs in 20 HPFs) was 13% in whole cases, and CE was positively correlated with the presence of endometriosis (*p* = 0.034). As this was a cross-sectional study without a control group, the prevalence in women without endometriosis was not reported. Contrary to the preceding studies [15,19], the prevalence of CE rose with the advancement of the stage of endometriosis (r = 0.302, *p* = 0.028). Qiao et al. [22] retrospectively identified the prevalence of CE in infertile women with minimal/mild endometriosis as 24.38%. The women with CE and minimal/mild endometriosis had a significantly lower cumulative clinical pregnancy rate (46.51% vs. 71.13%, *p* = 0.004) and live birth rate (44.19% vs. 63.38%, *p* = 0.025) compared with women without CE. Interestingly, the miscarriage rate in women with CE and minimal/mild endometriosis also tended to be lower than in women without CE but with minimal/mild endometriosis (0% vs. 7.04%, *p* = 0.074). The prevalence of CE in infertile cohorts without endometriosis was not reported here.

As many of these studies are of retrospective designs [14,15,16,17,18,20,22] and adopted different diagnostic criteria for IHC-CD138, caution should be exercised for the data interpretations. Moreover, the subjects or cohorts in the studies are biased toward women with operative indications for some gynecologic etiologies, like endometrioma, female infertility, and/or others. Thus, it is nearly impossible to know the general prevalence of CE in women with endometriosis, given the full requirements of laparoscopy, hysteroscopy, and endometrial biopsy for the diagnosis of two diseases. In addition, it remains unclear if the prevalence of CE in infertile women with endometriosis is higher than in those with other infertile etiologies, although relatively larger studies (enrolled 100 or more patients) reported that histopathologic CE is identified in 20.1% (55/273) of infertile women with polycystic ovarian syndrome and 19.8% (36/182) of infertile women with intrauterine adhesion [23,24]. The establishment of the unified diagnostic criteria to define the optimal threshold for the density of CD138(+) ESPCs are indispensable for data comparison among studies.

## 3. Local Inflammatory Profiling in Endometriosis and CE

### 3.1. Cellular and Humoral Immunology in Non-Pathologic Endometrium

Human endometrium contains a wide variety of leukocyte subpopulations. While one of the central roles of these mucosal leukocytes is the elimination of the endometrial cell debris that is shed during menstruation or accumulated products of concepts, they are also considered to serve for blastocyst implantation and placental development via the production of pro-inflammatory molecules, including chemokines and adhesion molecules that are essential for embryo migration, attachment, and deposition to the endometrium [25]. The density and proportion of endometrial leukocyte subpopulations drastically fluctuate across the menstrual cycle. Under a physiologic condition, the density of whole endometrial leukocytes is low in the proliferative phase and increases after ovulation, with a peak in the mid-to-late secretory phase and a spike in the menstrual period. Regarding the proportion, the leading leukocyte subpopulation in the proliferative phase is CD8(+) cytotoxic T cells, followed by CD4(+) helper T (Th) cells. Regulatory T cells are also seen in the endometrium, with a minor increase in the ovulatory phase. The density of these endometrial T cell subsets is almost constant throughout the menstrual cycle, whereas unique CD16^neg^ CD56^bright^ natural killer cells prominently rise after ovulation, along with an increase in macrophages and neutrophils in the mid-secretory phase. By contrast, the lineage of B cells is sparse in the endometrium regardless of the menstrual cycle [26]. Only a small number of B cells reside as the core cells of the lymphoid aggregates in the endometrial basal layer, surrounded by CD8(+) T cells and macrophages, and a few ESPCs are sporadically seen in 30% of women [27,28].

The expression of various subclasses of immunoglobulins (Igs), represented by heavy chains of IgG_1_, IgG_2_, IgM, IgA, IgM, light chains of Igκ, and J chain, has been identified in the nonpathological human endometrium [29,30]. IgA_1_ and IgA_2_ are constitutively expressed in endometrial surface and glandular epithelial cells, mainly at the apical side of cells as well as in the glandular lumina, implicating the pivotal role of these IgA subclasses in the front-line defense against foreign body invasion into the endometrial tissue, as seen in the other mucosal tissues. IgM is also expressed on the apical side of the endometrial surface and glandular epithelium, but some cells lack the expression. The expression levels of IgM and the proportion of cells that express IgM vary among individuals [30]. Most of the IgA- and IgM-bearing cells co-express the J chain and secretory component (polymeric Ig receptor), which are necessary for the generation of the polymeric Igs. The expression level of IgA_1_, IgA_2_, and IgM, along with the J chain and secretory component, in the endometrial epithelium is higher in the proliferative phase than in the secretory phase, suggesting the possible involvement of these Ig subclasses in embryo implantation and menstruation [29]. IgG_1_ and IgG_2_ are also present on the apical side of endometrial epithelial cells, with marked variances within and between individuals. Immunoreactivity to IgM, IgA_1_, IgA_2_, IgG_1_, and IgG_2_ is detectable more sparsely in the endometrial stromal compartment than in the endometrial epithelium. By contrast, IgG_3_, IgG_4_, IgE, and IgD are not detectable in the non-pathologic human endometrium [30].

The origin of these endometrial Ig subclasses remains fully unknown. It is thought that some monomeric IgA (lacking J chain) and IgG subclasses possibly enter the endometrial glandular epithelium by passive diffusion from the stromal compartment, but local pieces of machinery are present for an additional active external poly-Ig transport. Some of the endometrial glands express human leukocyte antigen-DR irrespective of the menstrual phase or degree of secretory component expression, suggesting active secretory component-mediated incorporation of serum-derived and locally produced polymeric Igs [29].

### 3.2. Cellular and Humoral Immunity in Eutopic Endometrium of Endometriosis

Based on their activated status and functions, macrophages are divided into two subtypes [31]. M1 macrophages (classically activated macrophages) are induced by stimulation of interferon-γ and promote local inflammatory responses via antigen presentation and production of interleukin (IL)-6, IL-12, and tumor necrosis factor (TNF)-α. On the contrary, M2 macrophages (alternatively activated macrophages) are differentiated by stimulation of IL-4 and inhibit local inflammatory responses via the production of IL-10, TNF-α, and arginase-I. Distinct from the non-pathologic endometrium, a postovulatory increase of macrophages is not observed in the eutopic endometrium of women with endometriosis. Instead, an unusual menstrual cycle-independent global augmentation of endometrial macrophages is seen in this pathology [32,33]. Uniquely, the density of local CD14(+)/CD68(+)/CD197(+)/CD80(+) M1 macrophages in the ectopic endometrium decrease with the progress of the disease, whereas that of CD14(+)/CD68(+)/CD163(+)/CD206(+) M2 macrophages increases from stage I to stage IV. These increases in eutopic endometrial M2 macrophages may play a role in the proliferation of ectopic endometrial cells and aberrant mucosal angiogenesis [34]. In parallel, the biases toward M2 macrophage are also observed in the ectopic endometrium of endometriosis [35,36]. The blocking of IL-6 with antagonistic antibodies was reported to reduce this shift from M1 macrophages to M2 counterparts, indicating the putative role of IL-6 in this alteration of local immune responses [35].

Meanwhile, the postovulatory rise of natural killer cells is maintained in the eutopic endometrium of women with endometriosis, but their cytolytic activity is impaired. In addition, the lowered activity of cytotoxic T lymphocytes, as well as the expansion of eosinophils, neutrophils, and mast cells, are reported in the peritoneal fluid in women with endometriosis [37]. Such an abnormal local immunological milieu is thought to allow the proliferation and survival of the ectopic endometrial tissues outside the uterus. Another cellular immunological feature of the eutopic endometrium of women with endometriosis is the appearance of ESPCs and CD20(+)/CD5(+)/human leukocyte antigen-DR(+) B cells [26,35], which are commonly found in the endometrium with CE.

As autoimmunity has been long believed to be associated with the onset and progress of endometriosis, studies sought autoantibodies against endometrial antigens in women with endometriosis. Although several studies reported that unusual IgG_1_ or IgG_2_ subclasses were detected in the serum of women with endometriosis, the entity of these antibodies remains elusive [38]. By contrast, endometrial Ig profiling in endometriosis remains yet undetailed. Early studies demonstrate that the local expression level of IgG subclasses is higher in the eutopic endometrium with endometriosis compared with those without endometriosis [38], but their subclasses were not described. Conversely, only a few studies investigated Ig profiling in the eutopic and ectopic endometrium with endometriosis. Early studies demonstrated that the local IgG expression level is higher in the eutopic endometrium with endometriosis compared with those without endometriosis [39]. In addition, IgG deposits accompanied by the C3 component of the complement cascade were found in the ectopic endometrioid tissues. These IgG subclasses in the eutopic and ectopic endometrium with endometriosis, however, were not described [40].

### 3.3. Cellular and Humoral Immunity in Endometrium with CE

It remains controversial if the density of the endometrial leukocyte subpopulations fluctuates in the endometrium with CE. Several studies did not report any differences from the non-pathologic endometrium [7,41], but others demonstrated an increase in the densities of whole macrophages, M2 macrophages, immature/mature dendritic cells, pan-T cells, and natural killer cells [42,43]. Regarding local Igs, the densities of endometrial IgM, IgA_1_, IgA_2_, IgG_1_, and IgG_2_ subclasses were found to be higher in women with CE than in those without CE, with the predominance of IgG2+ stromal cells [28]. We demonstrated the involvement of several chemokines (CXCL1 and CXCL13) and adhesion molecule (CD62E), which are abnormally expressed in endometrial endothelial and epithelial cells in women with CE, in the selective extravasation of peripheral blood B cells into the eutopic endometrium [7]. These pro-inflammatory molecules are locally induced by microbial antigens like lipopolysaccharide, the Toll-like receptor 2/4 ligand. Furthermore, the concentration of IL-6 and TNF-α is prominently higher in the menstrual blood of women with CE compared with those without CE [44,45].

Although it remains unelucidated if these hypotheses fully apply to the eutopic endometrium of endometriosis, studies support the idea that these unusual ESPCs and endometrial B cells are likely to play a role in the proliferation and survival of the endometrial cell components. For instance, the endometrium with local polyps and micropolyposis has proliferative characteristics and contains a larger number of ESPCs compared with the non-pathologic endometrium [46]. One of the histopathological characteristics of CE is a delay in the endometrial differentiation during the mid-secretory phase when blastocysts reach the uterine cavity and initiate the implantation process. We found that about one-third of the endometrium with CE displays an “out-of-phase” morphological appearance, including pseudostratification and mitotic nuclei in both glandular and surface epithelial cells [14]. Moreover, the expression of the gene transcripts associated with anti-apoptosis (*bcl2* and *bax*), nuclear division (*ki-67*), and ovarian steroid receptors (*esr1*, *esr2*, and *pgr*) are aberrantly elevated in the secretory phase endometrium with CE [47,48,49]. On the other hand, the expression of the gene transcripts potentially associated with embryo receptivity (*il11*, *ccl4*, *igf1*, and *casp8*) and decidualization (*prl* and *igfbp1*) are down-regulated in the endometrium with CE in this period [48,50]. These findings implicate that the endometrium with CE fails to respond adequately to ovarian steroids and to transform its component cells into a receptive phenotype that is indispensable for successful blastocyst implantation and placental development. These findings imply the potential relationship between CE and endometrial progesterone resistance, which is also observed in endometriosis [51].

In CE, the whole endometrial surface and glandular epithelium are immunoreactive to IgA_1_ and IgA_2_ [30]. Likewise, punctate or scattered immunoreactivity to IgA_1_ and IgA_2_ is identifiable in the endometrial stroma. On the contrary, the proportion of the endometrial glands immunostained for IgM varies among individuals (36.3–100%). IgG_1_ and IgG_2_ in the endometrial epithelium exhibit prominent intra-sample variances (57.1% and 42.9%, respectively). Moreover, the immunoreactivity to IgM, IgG_1_, and IgG_2_ is observed more sparsely in the endometrial stromal compartment than in the endometrial epithelium and differs in CE. The density of IgM+, IgA_1_+, IgA_2_+, IgG_1_+, and IgG_2_+ stromal cells is significantly higher in the CE than in the non-CE and control fertile women. In addition, the density of IgG_2_+ stromal cells is significantly higher than that of any other Ig subclass+ stromal cells. Sparse stromal immunostaining for IgG_3_ and IgE is occasionally seen in CE; immunostaining for IgG_4_ or IgD is not detectable [30]. These findings support the idea that local humoral immunity in the endometrium in CE is characterized as IgG_2_-dominant inflammation, like Crohn’s colitis. IgG_4_+ ESPCs are not identified in any of the endometrial samples [30], indicating that CE is unlikely to be associated with IgG_4_-related diseases, an intractable systemic pathologic entity with serum IgG_4_ elevation, tissue fibrosis, and infiltration of IgG_4_-bearing plasmacytes into various organs [52,53].

## 4. Reproductive Tract Microbiota in CE and Endometriosis

### 4.1. Reproductive Tract Microbiota in Healthy Women with Well-Being

Recent development and progress in next-generation sequencing enabled us to analyze the comprehensive microbial communities (microbiota) in various tissues and organs [54]. In 2011, the Human Microbiome Project, a research initiative led by the United States National Institutes of Health, demonstrated that the microbiota in the human vagina is dominated by four *Lactobacillus* species (*L. iners, L. crispatus, L. gasseri,* and *L. jensenii*), along with lower proportions of lactic acid bacteria. As it has been long indicated, the project disclosed the crucial role of lactic acid in the integrity of this organ [55]. By contrast, it remains to be determined if the *Lactobacillus*-predominant condition is beneficial for the integrity and maintenance of the upper female reproductive tract. In 2017, Chen et al. [56] fully investigated the microbiota in the upper and lower reproductive tract in Chinese women of reproductive age. They found that each reproductive organ has its unique microbiota, and the local microbiota in the reproductive organs is under the influence of multiple factors (age, body temperature, menstrual cycle, fecundability/infertility, and anemia).

### 4.2. Reproductive Tract Microbiota in Women with Endometriosis

The three major classical theories underlying the onset of endometriosis are (i) retrograde menstrual blood flow, (ii) coelomic metaplasia, and (iii) Mullerian remnants, but a single one cannot explain the whole entity of this multifactorial disease. Given the immunological and inflammatory backgrounds of the disease, it is conceivable that bacterial infections and their metabolites are associated with the etiology and pathogenesis of endometriosis [57].

Inconsistent results have been shown regarding the microbiota in the reproductive tract in women with endometriosis, particularly on *Lactobacillus* species. While some researchers reported a decrease in the proportion of *Lactobacillus* in the endometrial and vaginal microbiota with endometriosis [16], others found an increase [58,59,60] or invariance [61] from women without endometriosis. The possible explanations for these discrepancies among studies are (i) racial, ethnical, and geographical variances in the female reproductive tract microbiota [62], (ii) potential contamination of the bacteria from the tissue sampling devices (and/or of from the vagina, as aforementioned) [63,64], (iii) diversity in the disease stages and progress [65]. Thus, the bacterial genera/species and/or microbial communities in the female reproductive tract that are unique to endometriosis remain undetermined, and further studies are required.

Intriguingly, Khan et al. [19] demonstrated that the administration of gonadotropin-releasing hormone agonist, one of the therapeutic agents against endometriosis, possibly alters the microbiota in the uterine cavity, resulting in a further decrease in the proportion of *Lactobacillus* in Japanese women with endometriosis. Le et al. [66] and Chang et al. [67] also claimed that pelvic surgery and oral hormonal therapy potentially affect vaginal bacterial communities in women with endometriosis. For example, the proportion of *Lactobacillus* in the vaginal microbiota was lower in women using monophasic oral contraceptives than in the non-users. These findings support the idea that medical treatments other than antibiotic administration may have an impact on the reproductive tract microbiota in women with endometriosis, although it remains unclear if these local changes in microbial communities contribute to the suppression of the disease or the improvement of fecundity.

Meanwhile, Ata et al. [61] found an increase in the abundance of the pathogenic genera (*Gardnerella*, *Streptococcus*, *Escherichia*, *Shigella*, and *Ureaplasma*) in the cervical microbiota of Turkish women with advanced endometriosis. Interestingly, they reported a complete absence of *Atopobium* species, the potential pathogen in the female reproductive tract, in the vaginal and cervical microbiota in women with advanced endometriosis (stage III/IV). *Atopobium* is known to facilitate intracellular infection of *Porphyromonas*, which is a Gram-negative, non-spore-forming, obligately anaerobic, and non-motile bacterial genus that can disrupt cell regulatory functions and potentially trigger carcinogenesis in endometrial cells [65]. A decrease in *Atopobium* was also reported by another study [66]. The absence/reduction of *Atopobium* in the cervical microbiota is a compelling finding seen in endometriosis and awaits further studies. Chang et al. [67] reported that cervical microbiota in Taiwanese women with endometriosis might alter during disease development and progression. At the phylum level, there was a trend towards increased *Firmicutes* in combination with decreased *Actinobacteria* and *Bacteroidetes* in parallel with the advancement of endometriosis, the presence of deep-infiltrating endometriosis, severe dysmenorrhea, and infertility. At the genus level, increased *Lactobacillus* and *Streptococcus*, along with decreased *Dialister*, were frequently associated with advanced endometriosis. In addition, a significant reduction in the richness and diversity of the cervical microbiota was seen in patients with more severe clinical symptoms. Infertility treatments partially restored the eubiotic cervical microbiota in these women with advanced endometriosis via correction of nutrition and metabolism, molecular transport, and cell-cell/cell-matrix interaction. The members within the *Coriobacteriia* lineage (represented by *A. vaginae)* were the most common in the cervical microbiota in women without endometriosis, *L. jensenii* or members in *Corynebacteriales*, *Porphyromonadaceae*, and *Ruminococcaceae* were associated with women with stage I-II endometriosis, and *B. breve* and *Streptococcaceae* (like *Streptococcus agalactiae*) dominated in patients at stage III-IV. At the genus and species level, in contrast to *A. vaginae*, *Prevotella bivia*, and *P. amnii* being predominant in women without endometriosis, *R. erythropolis*, *P. gingivalis*, *and B. breve* were related to stage I-II and *S. agalactiae*, *P. bivia*, *and P. amnii* were to stage III-IV. *Lactobacillus* species other than *L. jensenii* did not show any differences among the groups.

Alternatively, Lu et al. [68] looked up the vaginal microbiota in women with and without endometriosis in Jiangxi Province, southeast China. Similar to previous studies, *Firmicutes* was most abundant, followed by *Actinobacteria*. However, distinct from the results by Chang et al. [67] on the cervical microbiota, they found a marked enrichment of *Actinobacteria* (particularly of *Gardnerella* and *Atopobium*), along with a prominent depletion of *Firmicutes* (particularly of *Lactobacillus*) in women with endometriosis compared with those without endometriosis. This local microbial composition pattern in endometriosis resembled that of bacterial vaginosis, a vaginal inflammatory disease characterized by elevated local pro-inflammatory cytokines and the impairment of the epithelial and mucosal barrier function. It is an enticing hypothesis if endometriosis develops with the breakdown of the vaginal bacterial composition, although further studies are required to elucidate it.

### 4.3. Reproductive Tract Microbiota in Women with CE

Literature to date shares some common or similar findings regarding the local microbiota in the reproductive tract in women with CE. For instance, several studies agree that several bacterial genera associated with bacterial vaginosis (*Gardnerella* and *Prevotella*) dominated the endometrial microbiota in CE (diagnosed based on the IHC-CD138) [69,70,71,72,73,74,75]. Bacterial vaginosis is a usual pathologic condition characterized by the reduction or deficiency of lactic acid-producing bacteria in the vaginal microbiome, along with increased anaerobic bacteria (*Gardnerella*, *Atopobium*, *Megasphaera*, *Prevotella*, and *Sneathia*).

Alternatively, a number of studies failed to detect any statistical differences in taxonomical composition and diversity in the genital microbiota between the CE and the non-CE group [76,77,78]. The bacterial load in the human vaginal cavity is estimated to be 100- to 10,000-fold higher than those in the cervical canal and uterine cavity [56]. No matter how local cleansing and disinfection are fully performed before tissue retrieval, the contamination of the vaginal bacteria into cervical/endometrial samples is inevitable in the process of the transvaginal procedure. The results of the cervical and endometrial microbiome analysis must thereby be interpreted with precautions. Indeed, the studies comparing the endometrial samples collected via the trans-peritoneo-myometrial route (during laparoscopy or laparotomy) and trans-vagino-cervical route disclosed quite different results on the local microbiota, particularly on the compositions of *Lactobacillus* species [54,65,66,67,68].

Fang et al. [70] first reported the endometrial microbiota in 25 infertile Southeast Chinese women with CE but without endometriosis using barcoded sequencing (V4 region) of the endometrial samples obtained by trans-vagino-cervical scraping on days 3–5 in the secretory phase. Their results were different from 69 infertile women without CE. While the most prevalent bacteria in the CE group were *Firmicutes*, followed by *Proteobacteria* and *Actinobacteria* at the phylum level, the most abundant one in the non-CE group was *Proteobacteria*, followed by *Firmicutes* and *Actinobacteria*; at the genus level, *Lactobacillus* dominated in the CE group, followed by *Enterobacter*, *Pseudomonas*, *Gardnerella*, and *Desulfosporosinus*, whereas *Enterobacter* was the most abundant in the non-CE group, followed closely by *Pseudomonas*, *Lactobacillus*, *Desulfosporosinus*, *Ralstonia*, and *Gardnerella*. Additionally, the proportion of *Enterobacter* and *Sphingomonas* was lower, and that of *Prevotella* was higher in the CE group than in the non-CE group. The potential bias of this study is that all women in CE had an endometrial polyp, which may affect the results.

Using endometrial biopsy and fluid collected in 7 days following luteinizing hormone surge and next-generation sequencing of 16S rRNA gene (V4 region), Liu et al. [71] investigated the local microbiota in 13 infertile women with CE, defined as the presence of more than 5.15 CD138(+) ESPCs/10 mm^2^ in the endometrial tissue, and 117 counterparts without CE who reside in the Hong Kong area. At the phylum level, *Firmicutes* was the most popular bacteria in the CE group, whereas *Actinobacteria* was the most abundant in the endometrial microbiota in the non-CE group, which was similar to the results by Fang et al. [70]. Likewise, at the genus level, *Lactobacillus* was the most abundant both in the CE group and also in the non-CE groups, but the relative abundance of *Lactobacillus* in the CE group was much (42.7 times) lower than that in the non-CE group. Conversely, *Dialister, Bifidobacterium*, *Prevotella*, *Gardnerella*, *Anaerococcus*, *Varibaculum*, *Howardella*, *Kocuria*, *Sphingomonadaceae*, *Corynebacterium*, *Tepidimonas*, *Micrococcus*, *Psychrobacter*, *Corynebacteriaceae*, *Peptoniphilus*, and *Luteimonas*, were more abundant, and *Acinetobacter* was detected exclusively in the CE group. At the species level, *L. iners* was prevalent in the CE group. By contrast, *L. crispatus* was scarce in the CE group, and four *Lactobacillus* species (*L. delbrueckii*, *L. coleohominis*, *L. mucosae*, and *L. antri*) that were detected constitutively in the non-CE group were absent in the CE group. Thus, the microbiota in CE was characterized by the relative increase in 18 bacterial genera in the uterine cavity. The correlation analysis revealed that *L. iners* had a negative correlation with *Anaerococcus*, *Finegoldia*, *Gardnerella*, *Polaromonas*, and *Staphylococcus*.

Lozano et al. [72] recruited 34 infertile Spanish women with CE (information unavailable on absence and concomitance of endometriosis), which was defined as two or more CD138(+) ESPCs in 5 HPFs (4 ESPCs in 10 HPFs) and 24 infertile women without CE for a comparison of the endometrial and vaginal microbiota (V3–V4 regions) in the secretory phase. At the phylum level, *Firmicutes* were the overwhelming majority in both CE and non-CE groups. At the genus level, the local microbiota in the non-CE group was represented with less diversity and predominance of *Lactobacillus*, that in the CE group was characterized by a decrease in *Lactobacillus* along with a rise in *Ralstonia* and *Gardnerella* in the endometrial microbiota as well as *Streptococcus* and *Ureaplasma* in the vaginal microbiota. The correlation analysis revealed that *Gardnerella* correlated with eight genera (positive correlation with *Anaerobacillus*, *Bacillus*, and *Ralstonia* and a negative correlation with *Dialister*, *Delftia*, *Burkholderia*, *Streptococcus*, and *Lactobacillus* in the endometrial microbiota. *Ralstonia* is a bacterial genus that belongs to *Burkholderiaceae*. A recent study demonstrated that the detection of *Ralstonia* and *Streptococcus* in the vaginal microbiota, accompanied by *Prevotella*, *Chlamydia*, *Bifidobacterium*, and *Aerococcus*, is associated with infection by human papillomavirus, the major cause of cervical cancer and intraepithelial neoplasia [73]. The findings suggest that these bacterial genera may contribute to maintaining and supporting local chronic infection in the female reproductive tract.

Chen P et al. [74] collected endometrial specimens in the 7 days following the luteinizing hormone surge in the natural cycles or 5 days following the progesterone supplementation in the artificial hormone cycle from 32 CE patients and 72 non-CE patients with a history of repeated implantation failure. *Phyllobacterium* and *Sphingomonas* were significantly enriched in the endometrial microbiota in the CE group. Both of these two genera were positively correlated with a local increase in multiple immunocompetent cell subpopulations (dendritic cells, natural killer cells, regulatory T cells, and B cells) except for macrophages which had a negative correlation. Moreover, signal pathway analysis revealed that endometrial microbiota in the CE group with abundant Th1 cells displayed several activated glycolysis-related pathways (super pathway of thiamine diphosphate biosynthesis I, reductive tricarboxylic acid cycle I, L-aspartate and L-asparagine biosynthesis, and purine nucleobases degradation I). Conversely, the PWY-7332 pathway (lipopolysaccharide synthesis) was highly active in the endometrium of CE patients with a high abundance of Th17 cells. Finally, they found that the main pathways activated in the CE group were the sucrose biosynthesis III (PWY-7347) and I (SUCSYN-PWY) pathways. Chen W et al. [75] included 94 infertile southeast Chinese women who were ready for their first IVF-ET cycle. Women with endometriosis were excluded. Twenty women (26.6%) were diagnosed with CE, which was defined as one or more plasma cells under one high-power field (HPF) and/or hysteroscopy. Endometrial fluid obtained at the same time was analyzed for microbiome analysis. The women identified with CE underwent a 14-day antibiotic treatment using ceftriaxone (250 mg/day, intramuscular), doxycycline (200 mg/day, oral), and metronidazole (800 mg/day, oral). They were subdivided into four groups according to the presence or absence of CE and the success or failure of clinical pregnancy (pregnant CE group, *n* = 8; non-pregnant CE, *n* = 17; pregnant non-CE, *n* = 41; and non-pregnant non-CE groups, *n* = 28). At the phylum level, *Proteobacteria* was the most dominant in the endometrial fluid in all groups, although its bacterial loads were significantly lower in the CE group than in the non-CE group and lower in the non-pregnant group than in the pregnant group regardless of the absence or presence of the history of CE. The same results went for *Acidobacteria*. On the contrary, *Actinobacteria* was significantly more abundant in the CE group than in the non-CE group and in the non-pregnant group than in the pregnant group. Interestingly, the bacterial loads of *Fusobacteria* were significantly higher in the pregnant CE group than in the other groups. At the genus level, *Lactobacillus* was the most dominant in all groups, followed by *Halomonas*, *Gardnerella*, and *Pelagibacterium*. The bacterial loads of *Gardnerella* were significantly higher in the CE group than in the non-CE group and were higher in the non-pregnant group than in the pregnant group, implicating the potential involvement of *Gardnerella* in CE and/or embryo implantation failure.

## 5. Pharmacotherapy against Endometriosis and CE

### 5.1. Antibiotic Treatment against Endometriosis

There is currently no literature that supports the effectiveness and safety of antibiotic treatment against endometriosis in humans. However, animal studies suggest the potential of some antibiotic agents in treatment modalities. Metronidazole is an imidazole derivative with an antibacterial and antiprotozoal activity that has been frequently prescribed against pelvic inflammatory disease, bacterial vaginosis, and CE (Table 2) [76].

Using the model mice with intraperitoneally implanted endometrial tissues, Chadchan et al. [81] examined the effects of 21-day oral administration of the combined water-solubilized antibiotic agents VNMA (an acronym for 0.5 mg/mL vancomycin, 1 mg/mL neomycin, 1 mg/mL metronidazole, and 1 mg/mL ampicillin) on endometriosis lesions. Of these antibiotic agents, metronidazole was demonstrated to significantly decrease the mass and weight of the ectopic endometriosis lesions, along with the correction of the pelvic inflammatory responses via suppression of macrophage proliferation and cytokine production including IL-1β, IL-6, and TNF-α. Interestingly, oral administration of feces obtained from mice with endometriosis reversed the growth of the pelvic endometriosis lesions and the development of the local inflammatory responses in the metronidazole-treated mice, indicating a key role of gut microbiota in the promotion and inhibition of endometriosis in mice. Lu et al. [68] also reported the effectiveness of 21-day (once every 3 days) vaginal administration of the VNMA mixture via an absorbable gel sponge on endometriosis lesions. The effect of VNMA treatment on endometriosis lesions with an intraperitoneal injection of the other antibiotic agent parthenolide (for times a week, for 3 weeks), which is capable of suppressing tissue proliferation and prostaglandin E2 production in the endometriosis lesions [82]. While the disorder of the vaginal microbiota potentially promoted the progression of endometriosis in this study, antibiotic treatment was capable of reducing the volume of the endometriotic lesions via regulation of the nuclear factor-kappa B signaling pathway [68].

Antibiotic treatment can be a potential therapeutic option against endometriosis in combination with other conventional treatments, although more basic studies are required before application to humans.

### 5.2. Antibiotic Treatment against CE

As a local infectious disease, antibiotic agents have been prescribed for infertile women with CE. Recent studies demonstrated that antibiotic treatments are superior to follow-up observations in the cure rate of histopathologic CE [83,84]. Moreover, several studies suggest that antibiotic treatments improved the live birth rate in subsequent embryo transfer cycles after the cure of histopathologic CE treatment [8,43,78,79,80,85,86,87], although there are no published randomized controlled studies.

Considering a wide variety of potential pathogens from common bacteria to *Mycoplasma*, broad-spectrum antibiotic agents, including oral doxycycline, fluoroquinolones (ofloxacin, levofloxacin, and ciprofloxacin), nitroimidazoles (tinidazole and metronidazole), have been prescribed in the treatment against CE [6,8,43,77,78,79,80,85,86,87,88]. Some studies adopted an antibiogram-oriented choice of antibiotic agents [8]. For infertile women with tuberculosis-associated CE, antitubercular chemotherapy based on a positive endometrial biopsy-polymerase chain reaction test improved their reproductive outcomes [89]. After 6-month administration of the antitubercular agents, the clinical pregnancy rate within 12 months was about 90%. Drug resistance against multiple antibiotic agents is, however, a serious global problem in the treatment of bacterial infectious diseases. CE is no exception anymore. We recently demonstrated an increase in multi-drug-resistant CE in infertile women with a history of repeated implantation failure. For example, in the year 2014, the resistance to the first-line oral doxycycline treatment (200 mg/day for 14 days) and the second-line combination of oral metronidazole (500 mg/day for 14 days) and ciprofloxacin (400 mg/day for 14 days) was less than 10% and 1%, respectively. By contrast, in the year 2020, the resistance to doxycycline exceeded 20%, and that to metronidazole/ciprofloxacin rose to 7.8% in whole CE cases [85].

There are several antibiotic agents that are still effective against multi-drug-resistant CE (e.g., azithromycin, moxifloxacin, and lincomycin) [85,86], but unnecessary prescriptions must be refrained to avoid the spread of multi-drug resistance against antibiotic treatment. To avoid the excessive administration of antibiotic agents, some hormonal treatments, which have been traditionally utilized against endometriosis, have been attempted against CE. Only a few well-defined studies reported that some hormonal agents (dydrogesterone, GnRH agonists, etc.) display the additive effects of antibiotic treatment on CE [19,90]. These findings may be worth reevaluating in well-designed and larger settings.

## 6. Commonalities and Disparities between Women with Endometriosis and CE

Studies demonstrated that endometriosis and CE share some common immunologic backgrounds. For example, unusual infiltration of B cells and ESPCs within the endometrium has been documented in both diseases, along with increased local production of several proinflammatory cytokines, such as IL-6 and TNF-α. IL-6 is known as a differentiation factor of immature B cells into mature B counterparts in various tissues, whereas TNF-α is capable of stimulating the local biosynthesis of estrogens in endometrial glandular cells, which potentially transforms endometrial cells into the proliferative phenotype that may cause the occurrence of endometrial micropolyposis, the tiny protrusive lesions often identified in women with CE under fluid hysteroscopy [46,47]. In parallel, higher levels of expression of some Ig subclasses (IgG_1_, IgG_2_, and possibly IgA) have also been detected commonly in the eutopic endometrium with endometriosis and CE, suggesting the upregulation of Ig production by infiltrating ESPCs [28,30]. These elevated inflammatory responses may potentially relate to the progress and development of both diseases.

By contrast, the results of microbiome analyses are inconsistent and sometimes conflicting among the studies. These discrepancies are likely to originate in the races, ethnicities, and sampling sites/procedures. One unique microbial finding in endometriosis is the absence/reduction of *Atopobium* in the cervical microbiota, although its significance in this pathology remains unknown [65,66]. These findings warrant further investigation into the disparities between the two diseases.

Animal studies indicate the therapeutic potential of some antibiotic agents against endometriosis, particularly metronidazole, which has been prescribed against CE [6,43,77,78,79,80,81,82]. As the effectiveness and safety of antibiotic treatment against endometriosis remain to be investigated in humans, thus well-designed studies are essential before clinical application.

## 7. Conclusions

While endometriosis has been long considered a cause of infertility, CE is also an emerging issue that may reduce fecundity in women of reproductive age. While endometriosis and CE share features of endometrial proliferative nature, these two mucosal diseases have some different characteristics. Scientific approaches to these commonalities and disparities in immunological backgrounds between endometriosis and CE may lead to the distinction between the women with CE who potentially develop into endometriosis and who do not. The potential relationships between endometriosis and CE warrant future studies.

## Figures and Tables

**Table 1:** Publications on the prevalence of histopathologic CE (diagnosed according to IHC-CD138) in women with or without endometriosis.

Authors [Reference]/ Published Year/Nation/ Study Period/Study Design	Reported Prevalence of CE in Endometriosis Group and (*p*-Value)	Age (Years)	BMI (kg/m^2^)	Sample Source	Conditions for IHC-CD138	Adopted Diagnostic Criteria for CE	Stage of Endometriosis and Relationship with CE *
Kitaya K et al. [14]/ 2011/Japan/ January 2002–December 2010/ retrospective study	5.00% (1/20, endometriosis group) vs. 11.68% (25/214, non-endometriosis, endometrial benign diseases) (*p* = 0.7072)	Information unavailable	Information unavailable	Hysterectomy	Paraffin-embedded 4-μm sections/clone B-A38 (Nichirei Corp., Tokyo, Japan), stock solution, 60 min, room temperature	5 or more ESPCs in 10 high power fields (HPFs) (400-fold magnification)	Information unavailable
Takebayashi A et al. [15]/ 2014/Japan/ April 2001–December 2012/ retrospective study	52.94% (18/34, endometriosis group) vs. 27.02% (10/37, non-endometriosis, endometrial benign diseases) (*p* = 0.0311)	44.15 ± 3.65 vs. 43.15 ± 2.75 (mean ± SD)	22.08 ± 4.83 vs. 21.60 ± 3.14 (mean ± SD)	Hysterectomy	Paraffin-embedded 4-μm sections/B-A38, stock solution, 60 min, room temperature	1 or more ESPCs in 10 HPFs (400-fold magnification)	Stage I-IV, no relationship between the prevalence of CE and stage
Khan KN et al. [16]/ 2014/Japan/ June 2012–December 2013/ retrospective study	3.08% (2/65, endometriosis, infertility/dysmenorrhea group) vs. 0% (0/55, non-endometriosis, infertility/dysmenorrhea) (*p* = 0.4993)	21–51 vs. 22–51 (range)	Information unavailable	Endometrial curettage	Paraffin-embedded 5-μm sections/clone ab34164 (Abcam, Tokyo, Japan), 1:200 dilution, overnight, 4 °C	1 or more ESPCs in 15 HPFs (100-fold magnification) in 3 or more sections	Information unavailable
Cicinelli E et al. [17]/ 2017/Italy/ January 2010–June 2016/ retrospective study	38.46% (30/78, endometriosis group) vs. 14.10% (11/78, non-endometriosis, endometrial benign diseases) (*p* < 0.001) *	44.3 ± 2.8 vs. 44.0 ± 2.3 (mean ± SD)	27.3 ± 4.2 vs. 27.2 ± 4.3 (mean ± SD)	Hysterectomy	Paraffin-embedded 4-μm sections/clone MI15 (Cell Marque Biocare Medical, Concord, CA)/not available	1 or more ESPCs in 10 HPFs (100-fold magnification)	Stage IV
Freitag N et al. [18]/ 2020/Germany (>90% Caucasian)/ January 2013–February 2017/ retrospective study **	12.90% (8/62, endometriosis group, infertility) vs. 10.00% (5/50, non-endometriosis, infertility) (*p* = 0.634)	26–48 (range)	Information unavailable	Pipelle suction	Paraffin-embedded/ other information not available (sent to laboratory)	5 or more ESPCs per mm^2^ section	Information unavailable
Khan KN et al. [19]/ 2021//Japan/ April 2015–February 2017/ prospective non-randomized study	≥22.6% (≥12/53) Not examined prior to treatment 33.4% (7/21) (Untreated endometriosis) ≥23.4% (≥11/47) Not examined prior to treatment 27.3% (3/11) Untreated endometriosis	18–51 vs. 26–51 (range)	Information unavailable	Endometrial curettage	Paraffin-embedded 5-μm sections/ab34164, 1:200 dilution overnight, 4 °C	1 or more ESPCs in 5 HPFs (200-fold magnification)	Stage I-IV, no relationship between the prevalence of CE and stage

* According to the Revised American Society for Reproductive Medicine classification [20]. ** The bias of this study is that some women (the number not detailed in the article) did not take laparoscopy or laparotomy and were regarded as having endometriosis only by symptomatology.

**Table 2:** Studies on the use of metronidazole against CE.

Authors [Reference]/ Published Year/Nation/ Study Period/Study Design	Dose	Indications	Age (Years)	BMI (kg/m^2^)	Conditions for IHC-CD138	Diagnostic Criteria for CE	The Cure Rate of Histopathologic CE
Johnston-MacAnanny EB et al. [6]/ 2010/USA/ January 2001–December 2007/ retrospective study	1000 mg/day, 14 days (500 mg, twice) in combination with ciprofloxacin 1000 mg/day, 14 days	Repeated implantation failure (two failed ET cycles), second-line treatment against doxycycline-resistant CE	34.5 ± 3.27 (mean ± SD)	Information unavailable	Pipelle suction specimens/Immunohistochemistry, paraffin-embedded sections/MI15 Cell Marque (Biocare Medical, Concord, CA)/not available Biocare Medical, Concord, CA)/1:100 dilution/60 min/Room air?	1 or more ESPCs in 1 HPF observed	100% (3/3)
McQueen DB et al. [43]/ 2014/USA (Caucasian and African-American)/ July 2004–February 2012/ prospective study	1000 mg/day, 14 days (500 mg, twice) in combination with or ofloxacin 800 mg/day, 14 days	Recurrent pregnancy loss, first-line treatment	22.08 ± 4.83 (mean ± SD)	25.8 ± 6.4, and 20–47 (mean ± SD, and range)	Not detailed	Not detailed	73.1% (19/26)
Yang R et al. [77]/ 2014/China /January 2009–January 2010/prospective study	1000 mg/day, 14 days (500 mg, twice)in combination with levofloxacin 500 mg/day, 14 days	Repeated implantation failure (three failed ET cycles or 6 or more high-quality transferred embryos), first-line treatment	Not detailed (Two combined studies are reported in one article)	Not detailed (Two combined studies are reported in one article)	Pipelle suction specimens/Immunohistochemistry	1 or more ESPCs in the section observed	Not re-examined (?/68)
Tersoglio AE et al. [78]/2015/Argentina/ 2010–2013/ prospective study	1000 mg/day, 14 days (500 mg, twice)in combination with ciprofloxacin 1000 mg/day, 14 days and precedent 200 mg/day doxycycline along with prednisone 4–8 mg/day	Repeated implantation failure (two or more failed ET cycles) first-line treatment	36.0 ± 4.08 (mean and SD)	Information unavailable	Not detailed	1 or more ESPCs in 1 HPF observed	64.3% (9/14)
Kitaya K et al. [79]/ 2017/Japan/ November 2011–July 2014/ prospective study	500 mg/day, 14 days (250 mg, twice) in combination with ciprofloxacin 400 mg/day, 14 days	RIF (three or more 6 or more high-quality transferred embryos and/or blastocysts), second-line treatment against doxycycline-resistant CE	38.1 ± 3.8 (mean ± SD)	21.1 ± 1.9 (mean ± SD)	Curette biopsy specimens/ Immunohistochemistry, paraffin-embedded 4-m sections/B-A38 (Nichirei Corp., Tokyo, Japan), stock solution, 60 min, room temperature	endometrial stromal plasmacyte density index (sum of ESPC counts divided by the number of HPF evaluated) 0.25 or more	88.9% (8/9)
Gay C et al. [80]/ 2021/France/ January 2013–January 2018/ retrospective study	1000 mg/day, 14 days (500 mg, twice) in combination with doxycycline 200 mg/day, 14 days (Antibiotic agents were chosen according to antibiogram if bacteria were identified.)	Recurrent pregnancy loss, first-line treatment	33 and 9 (median and interquartile range)	24 and 3 (median and interquartile range)	Pipelle suction specimens/Immunohistochemistry, not detailed	1 or more ESPCs in 1 HPF observed	100%? Not detailed

## Data Availability

Not applicable.
